# HIV-Infected or -Exposed Children Exhibit Lower Immunogenicity to Hepatitis B Vaccine in Yaoundé, Cameroon: An Appeal for Revised Policies in Tropical Settings?

**DOI:** 10.1371/journal.pone.0161714

**Published:** 2016-09-22

**Authors:** Anne Esther Njom Nlend, Philippe Salomon Nguwoh, Christian Taheu Ngounouh, Hyppolite Kuekou Tchidjou, Constant Anatole Pieme, Jean Mbede Otélé, Véronique Penlap, Vittorio Colizzi, Roger Somo Moyou, Joseph Fokam

**Affiliations:** 1 Pediatric Service, National Insurance Fund Welfare Hospital, Yaoundé, Cameroon; 2 Faculty of Medicine and Biomedical Sciences, University of Yaoundé 1, Yaoundé, Cameroon; 3 Higher Institute of Health Professions, Yaoundé, Cameroon; 4 Ministry of Public Health, Yaoundé, Cameroon; 5 Institute of Science and Technology Applied to Health, Yaoundé, Cameroon; 6 Bambino Gesu Paediatric Hospital, Rome, Italy; 7 Department of Biochemistry and Microbiology, Faculty of Science, University of Yaoundé 1, Yaoundé, Cameroon; 8 Chantal BIYA International Reference Centre for Research on HIV/AIDS prevention and management, Yaoundé, Cameroon; 9 University of Rome Tor Vergata, Rome, Italy; 10 UNESCO Interdisciplinary Board of Biotechnology, Rome, Italy; 11 Institute of Research in Medicine and Medicinal plants, Yaoundé, Cameroon; Asociacion Civil Impacta Salud y Educacion, PERU

## Abstract

**Background:**

Since 2005, anti-hepatitis B virus (anti-HBV) vaccine is part of the Expanded Program on Immunization (EPI) for infants born in Cameroon, with 99% anti-HBV coverage. In a context of generalized HIV epidemiology, we assessed paediatric anti-HBV vaccine response according to HIV status, feeding option and age in a tropical context.

**Methodology:**

Prospective, observational and cross-sectional study conducted among 82 children (27 [IQR: 9–47] months, min-max: 6–59), after complete anti-HBV vaccination (Zilbrix Hepta: 10μg AgHBs) at the Essos Health Centre in Yaounde, Cameroon, classified as group-A: HIV unexposed (28), group-B: HIV-exposed/uninfected (29), group-C: HIV-infected (25). Quantitative anti-HBs ELISA was interpreted as “no”, “low-” or “protective-response” with <1, 1–10, or ≥10 IU/L respectively; with p-value<0.05 considered significant.

**Results:**

Children were all HBV-unexposed (AcHBc-negative) and uninfected (HBsAg-negative). Response to anti-HBV vaccine was 80.49% (66/82), with only 45.12% (37/82) developed a protective-response (≥10IU/L). According to HIV status, 60.71% (17/28) developed a protective-response in group-A, vs. 51.72% (15/29) and 20% (5/25) in group-B and group-C respectively, Odds Ratio (OR): 2.627 [CI_95%_ 0.933–7.500], p = 0.041. According to feeding option during first six months of life, 47.67% (21/45) developed a protective-response on exclusive breastfeeding vs. 43.24% (16/37) on mixed or formula feeding, OR: 1.148 [CI_95%_ 0.437–3.026], p = 0.757. According to age, protective-response decreased significantly as children grow older: 58.33% (28/48) <24 months vs. 26.47% (9/34) ≥24 months, OR: 3.889 [CI_95%_ 1.362–11.356], p = 0.004; and specifically 67.65% (23/34) ≤6 months vs. 0%, (0/5) 33–41 months, p = 0.008.

**Conclusions:**

Anti-HBV vaccine provides low rate of protection (<50%) among children in general, and particularly if HIV-exposed, infected and/or older children. Implementing policies for early vaccination, specific immunization algorithm for HIV-exposed/infected children, and monitoring vaccine response would ensure effective protection in tropical settings, pending extensive/confirmatory investigations.

## Introduction

### Epidemiology of HBV and HIV in sub-Saharan Africa

Viral hepatitis B (HBV) and C are globally known as prevailing agents in hepatocellular carcinoma (HCC) and associated mortality [1]. As the main cause of chronic liver infection, most of the 400 million HBV-infected individuals worldwide are found in African and Asian countries, with highly endemic prevalence (8–15%) [1,2]. Within paediatric populations, vertical transmission of HBV is more frequent in Asia, against horizontal transmission (unsafe household contacts, scarification, or medical interventions) in sub-Saharan Africa (SSA). As early age HBV-infection is associated with risks of chronic infection (90%) and HCC (as high as 50%), children in higher endemic settings, such SSA, deserve special considerations [2,3].

Out of 36.7 million of HIV-infected individuals (including 2.6 million children) worldwide, ~70% are living in SSA, with consistent risks of mother-to-child transmission [4,5]. Furthermore, the high prevalence of HIV-infection during pregnancy in Africa (7.8% in Cameroon) suggests considerable rates of HIV-vertical exposure, in the frame of continuous risks of mother-to-child transmission [6]. The prevalence of HIV-1 among infants from the prevention of mother to child transmission (PMTCT) is 11.5% (434/3789) in Cameroon [7], suggesting eventual impaired T-cell immunity and inferior response to vaccination in these potentially vulnerable populations [8,9].

### HBV vaccination in African children

In HBV-endemic settings like SSA, anti-HBV vaccination is strongly recommended to every newborn [10,11]. Of note, the high rate of paediatric HBV-exposure (19.4%) in certain central African countries supported the integration of anti-HBV vaccine into the Expanded Program on Immunization (EPI) since 2005 in most of these countries, with an estimated coverage of 99% in Cameroon [12,13]. Therefore, to sustain the current low paediatric HBV-infection (0.7%, favoured by low maternal HBeAg) in a country with high burden of HIV/HBV coinfection [12–15], ensuring decent protection of children would be relevant [11].

### Paediatric HBV vaccine response

In contrast to the high vaccine-induced HBV-protection rate (90–95%) observed among children living in western countries [16], children from several SSA countries have been experiencing suboptimal protection after anti-HBV vaccination (42.9–68.0% in Cameroon) [13,17,18], indicating a possible need of a booster dose to achieve effective immunization [19]. However, little is known on factors underpinning such poor response in such settings, among which HIV-vertical exposure. Interestingly, and in contrast to other EPI common vaccines (BCG, Hib, pertussis, tetanus), HIV-exposed infants might have a significantly lower response to anti-HBV vaccine compared to their HIV-uninfected pairs [20]. Furthermore, compared to HIV uninfected children, HIV-infected ones might be at higher risk of HBV-infection without an adapted vaccine schedule (15.8% versus 61.1% vaccine response in Kwazulu-Natal, respectively) [21]. This underscores the necessity for HIV-infected children, with no or mild immunosuppression, to eventually receive a double dose schedule of HBV vaccine to achieve protection against HBV [22]. Translating these findings in a context of HIV-vertical exposure and/or infection foresees a lower probability of developing a protective vaccine-induced response in such vulnerable children [20–22]. Moreover, HIV-vertically exposed children would be more vulnerable to HBV acquisition through viral chromosomal synthesis in the form of covalently closed circular DNA (cccDNA) in hepatocytes, herein justifying challenges in HBV cure/eradication with current antivirals as well as the need for optimal protection of vulnerable ones [18,23].

In a tropical context with highly prevalent HIV/HBV coinfection and consistent MTCT like Cameroon [6,7,14], investigations aiming at evaluating paediatric anti-HBV vaccine response, including a comparative analysis with HIV-exposed/infected children, would delight previous findings. Furthermore, such evidence would provide an added value on monitoring anti-HBV vaccine response in the context of paediatric HIV exposure and/or infection in Cameroon, by identifying children requiring a booster dose for lifelong protection against HBV [17,19,22].

### Study objectives

We herein sought to ascertain the overall post-vaccination response against HBV in children, compare the degree of HBV protection according to HIV vertical exposure and infection, evaluate the impact of infant feeding option and age on vaccine response.

## Materials and Methods

### Study design and target population

A prospective, observational and cross-sectional study was conducted from 05^th^ May– 04^th^ August in 2014 among HBV-vaccinated children at the Essos Health Centre (EHC) in Yaounde, Cameroon; stratified according to HIV profile, feeding option and age range.

### Description of paediatric anti-HBV prevention strategy in the target population

EHC is a reference health facility based in the capital city of Cameroon, a township of 180 km^2^ featuring a tropical wet and dry climate with constant temperature (22.9–25.7°C mean daily temperature); the population is over 2.5 million inhabitants, for a density of 14,000/km^2^. EHC currently has a large accommodation capacity of ~250 beds, innovative technical equipment and a very high-performance within a wide range of specialised services: general medicine, emergency, anaesthesiology, reanimation, surgery and sub-specialities, gynaecology, ophthalmology, dentistry, medical imaging, laboratories (including molecular biology), pharmacy, neonatology, paediatrics, etc. Specifically, EHC is a reference antiretroviral treatment (ART) centre for both adults and children since 2005, with ~450 HIV-infected children on ART to date. This performance ranked the EHC as the four national reference center for paediatric ART in Cameroon.

At the EHC, PMTCT of HBV at the study site follows national guidelines, and starts with antenatal screening for HBV infection for all pregnant women during antenatal care. For positive-HBsAg mothers, early neonatal administration of anti-HBV immunoglobulin is strongly recommended within 48 hours after birth. Both for HBV vertically exposed and unexposed infants, anti-HBV vaccine (Zilbrix Hepta) is administered (0.5ml IM at the anterolateral aspect of the thigh, containing 10μg of AgHBs) at a series of three monthly doses. Assessing antibody response consists of anti-HBs serological testing recommended at least 1–4 months after the final (third) vaccine dose.

### Sampling method and enrolment

Based on a convenient sampling, the sample size was calculated using the following formula:

$$\text{N}=\frac{{\text{Z}}^{2}\times \text{P}\left(1-\text{P}\right)}{{\text{D}}^{2}}$$

With “Z” being 1.645 at 95% confidence interval (CI), “P” being the previous rate of anti-HBV vaccine response in the same city (42.9%) [11], and “D” being the error rate set at 9% (0.09); resulting in a minimum sample size “N” = 81.8, rounded-up to 82 study participants.

At the vaccination unit of the EHC paediatric service, mothers and their children attending the routine immunization program were provided with information on the study objectives. Then, 200 mothers of potentially eligible children received the written information sheet and the study informed consent form. Of these 200 women, 34% (88) mothers returned with a signed informed consent form, and their children were enrolled. Vaccination charts of the 88 children were then checked by retrieving information to confirm eligibility to study criteria, mainly based on age (6–59 months), on complete anti-HBV vaccination (all 3 vaccine doses), and on the absence of advanced stage of immunodeficiency (CD4<200 cells/mm^3^) for HIV-infected children. Four children had received the third vaccine dose ≥4 months ago prior to enrolment, while two others had insufficient samples for laboratory analysis. Final sampling therefore had 82 study participants. The HIV status of the 82 participants, as well as the CD4 count of HIV-infected children, was confirmed from their medical records at EHC.

As per the study primary outcomes, participants were classified into three groups following their HIV profile: HIV unexposed (group-A), HIV-exposed but uninfected (group-B), and HIV-exposed and infected (group-C).

As per the study secondary outcomes, participants were furthered stratified according to feeding option and age in the frame of anti-HBV vaccine response.

### Testing for HBV biomarkers

Evaluation of post-vaccination response was based on a component of three different HBV-specific biomarkers, among which anti-HBs, anti-HBc and HBsAg tests.

#### Anti-HBs test

Levels of anti-HBs antibody titer was measured to determine the threshold of effective protection in HBV vaccinated children, using the quantitative anti-HBs Sandwich enzyme-linked immunosorbent assay (ELISA) of CTK Biotech, USA (lot: E0604K3), as per the manufacturer’s instructions. Briefly, reagents were brought at room temperature (18–25°c), wash solutions were prepared with phosphate buffer saline (1/30), and 50 μl of controls (negative and positive) and samples were added in respective wells. 50 μl of conjugate was added in each well (except for the blank), followed by incubation at 37°c for 60 min. Wells were washed and 50 μl of substrates A and B added in every well (including the blank), then incubated for 30 min at room temperature in a back room. 100μl of stop solution was then added to each well and reading of optical densities performed at 450 nm wave length, with reference to 630–690 nm. Assay validation was performed with optical density of ≤ **0.10** for the mean negative control and of ≥0.80 for the mean positive control. Cut-off value was calculated using the mean negative control x 2.1. Samples were considered Anti HB_S_ positive if D.O ≥1.00, Anti HB_S_ negative or “no vaccine response” if D.O <1.00. “Protective response” was defined as Anti HB_S_ titer >10 UI/ml; “low response” as Anti HB_S_ titer: 1–9 UI/ml; and “no response” Anti HB_S_ titer <1 UI/ml.

This anti-HBs ELISA kit is coupled to the systematic detection of anti-HBc marker, irrespective of anti-HBs present or absent in the analysed serum (CTK Biotech, USA).

#### Anti-HBc test

Anti-HBc marker was used to qualitatively detect any child with past/on-going exposure with HBV. In addition to aforementioned testing of anti-HBc, anti-HBc was also tested using the lateral flow chromatographic immunoassay HBV-5 Rapid Test (CTK^®^, USA), with 50μl of serum. Validated results were reported as Anti-HBc “response” or “no response”. Both Anti-HBc results were all concordant, confirming HBV unexposed status.

#### HBsAg test

HBsAg marker was used to qualitatively detect any child infected with HBV, using lateral flow chromatographic immunoassay HBSAg Gold Rapid Screen (collodial gold) of BioSino Biotechnology & Science Inc (Beijing, China), with 100μl of serum. Validated results were reported as HBsAg reactive (HBV-positive) or non-reactive (HBV-negative).

#### Data interpretation of study biomarkers

Vaccine response was defined by a profile of anti-HBs positive, anti-HBc negative and HBsAg negative; further stratified as “protective-response” (>10 UI/ml anti-HB_S_) “low-response” (1–9 UI/ml anti-HB_S_), or “no response” (<1 UI/ml anti-HB_S_).

### Data collection and statistical analysis

Data were collected using a standard questionnaire, then abstracted in a MS excel spreadsheet. Data were then entered into, and analysed with, Epi Info software v.3.5.3. Statistical association between anti-HBV vaccine response and children characteristics (HIV exposure and status, feeding option, and age) were performed using Chi-square and Fisher Exact test where appropriate, with corresponding 95% CI. P-values, generated by SAS, SPSS, R software, were considered statistically significant if p<0.05.

### Ethical considerations

Administrative authorisation (Reference: N°005/14/DCHE/DA/CE/CHE/CNPS) was issued and ethical clearance for the study obtained from the Institutional Review Board (IRB) of the Essos Health Centre (Reference: N°2014/001/EC-CHE). Mothers of potential participating children received detailed information on the study (with provision of an information sheet). Compliant mothers then provided each a signed written proxy-informed consent; this consent procedure was approved by the IRB. Laboratory results were freely offered for participant clinical benefits, data management was under strict confidentiality by using specific identifiers and restricted access.

Study dataset is provided as supporting information (S1 File).

## Results

### Characteristics of study participants

In total, 82 eligible children were enrolled in the study, all being anti-HBc and HBsAg negative. Sex ratio was similar (F/M: 5/4), median [interquartile range, IQR] age was 27 [IQR: 9–47] months, min-max: 6–59 months. According to maternal HBV status, 22 children were born to HBV-negative mothers (of whom 7 were HBV-vaccinated), four from HBV-positive mothers, and 56 from mothers with unknown HBV-status.

All HIV-infected children were normal clients (none hospitalized) attending the routine immunization program; all were receiving antiretroviral therapy and the majority were asymptomatic without any event of advanced immunodeficiency (Table 1).

**Table 1:** Basic demographic and clinical characteristics of children enrolled in the study. NA: not applicable; WHO: World Health Organization; IQR: interquartile range.

Group category (number)	Group-A (28)	Group-B (29)	Group-C (25)
**History of HIV vertical exposure**	HIV-unexposed	HIV-exposed	HIV-exposed
**Status of HIV infection**	HIV-negative	HIV-negative	HIV-positive
**Median age, [IQR], (min-max) months**	18 [9–46], (6–59)	13 [9–29], 6–58	40 [13–57], 6–59
**WHO clinical classification stage**	NA	NA	I (64%) vs. II (36%)
**Median CD4, [IQR], (min-max) cells/mm^3^**	NA	NA	375 [304–460], 257–1115

According to feeding option of the 82 children during the first six months of life, 54.87% experienced exclusive-breastfeeding, 26.83% formula feeding, and 18.30% mixed feeding. All these children were not exposed to HBV, reported by a negative result both to anti-HBc and HBsAg testing.

### Overall response to anti-HBV vaccine

Overall assessment of anti-HBV post-vaccine response showed that: 80.49% (66/82) of children responded to anti-HBs testing (positive anti-HBsAb), indicating an immune response following anti-HBs vaccination, while 19.51% (16/82) did not responded to vaccination (negative anti-HBsAb).

Analysis on the level of protective vaccine response revealed that only 45.12% (37/82) developed a protective immune response against HBV (anti-HBsAb ≥10IU/L) against 35.37% (29/82) who developed a low post-vaccine response.

### HBV post-vaccine response according to HIV vertical exposure

In group-A (HIV unexposed children), 82.14% (23/28) responded to HBV-vaccination and 60.71% (17/28) had a protective response. In the group of HIV-vertically exposed children, 79.63% (43/54) responded to HBV-vaccination and only 37.03% (20/54) had a protective response. Compared to HIV-unexposed children, the level of anti-HBV protection dropped significantly (~two fold) in HIV-exposed children as compared to (60.71% versus 37.03%, respectively, Odds ratio (OR): 2.627 [CI_95%_ 0.933–7.5], p = 0.041), as shown in Table 2.

**Table 2:** Anti-HBV vaccine response in HIV-exposed vs. HIV-unexposed children. In red are proportions of children with a protective-response after anti-HBV vaccination in each category.

HIV EXPOSURE STATUSANTI-HBV RESPONSE (HBsAb titer)	HIV-vertically exposed children (18 [9–47] months old)		HIV-vertically unexposed children (18 [9–46] months old)	
	N	%	N	%
**No response (< 1UI/L)**	**11**	**20.37**	**5**	**17.86**
**Low-response (1UI/L—9UI/L)**	**23**	**42.60**	**6**	**21.43**
**Protective-response (≥10UI/L)**	**20**	**37.03**	**17**	**60.71**
**TOTAL**	**54**	**100**	**28**	**100**

### HBV post-vaccine response in HIV-exposed/uninfected versus HIV-exposed/infected

Among HIV-exposed/uninfected children, 82.76% (24/29) responded to HBV-vaccination and 51.72% (15/29) had a protective response. Among HIV-exposed/infected children, 76.00% (19/25) responded to HBV-vaccination and only 20.00% (5/25) developed a protective response. Compared to HIV-exposed/uninfected children, the level of anti-HBV protection dropped in HIV-exposed/infected children (51.72% versus 20.00%, respectively, OR: 4.286 [CI_95%_1.099–17.602], p = 0.065).

### HBV post-vaccine response in HIV-infected versus all HIV-uninfected

Among HIV-infected children, 76.00% (19/25) responded to HBV-vaccination and only 20.00% (5/25) had a protective response. Among all HIV-uninfected children (exposed or unexposed), 82.46% (47/57) responded to HBV-vaccination and 56.14% (32/57) had a protective response. Compared to HIV-uninfected children, the level of anti-HBV protection dropped significantly (~three fold) in HIV-infected children (51.72% versus 20.00%, respectively, OR: 5.120 [CI_95%_ 1.521–18.252], p = 0.002), as shown in Table 3.

**Table 3:** Anti-HBV vaccine response in HIV-infected vs. HIV-uninfected children. In red are the highest rates of response after anti-HBV vaccination in each category of children.

HIV INFECTION STATUSANTI-HBV RESPONSE (HBsAb titer)	HIV-infected children (40 [13–57] months old)		HIV-uninfected children (15 [9–37] months old)	
	N	%	N	%
**No response (< 1UI/L)**	**6**	**24.00**	**10**	**17.55**
**Low response (1UI/L- 9UI/L)**	**14**	**56.00**	**15**	**26.31**
**Protective response (≥ 10UI/L)**	**5**	**20.00**	**32**	**56.14**
**TOTAL**	**25**	**100.00**	**57**	**100.00**

### Impact of breastfeeding option on anti-HBV post-vaccine response

**Fig 1:**
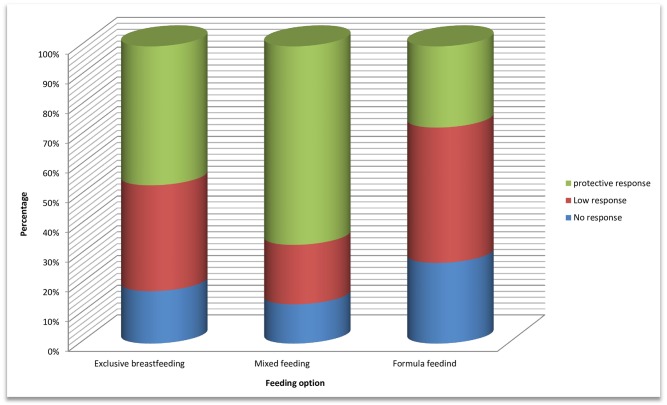
Impact of feeding option on anti-HBV vaccine response. “Protective response” (Anti HB_S_ titer >10 UI/ml); “Low-response” (Anti HB_S_ titer 1–9 UI/ml); “No response” (Anti HB_S_ titer <1 UI/ml).

Evaluation of vaccine efficacy according to feeding options during first six months of life revealed that protective immune response was found in 47.67% (21/45) on exclusive breastfeeding, against 43.24% (16/37) on mixed (15) or formula feeding (22), OR: 1.079 [CI_95%_ 0.639–1.869], p = 0.757 (Fig 1).

### Variability of paediatric anti-HBV response with age

**Fig 2:**
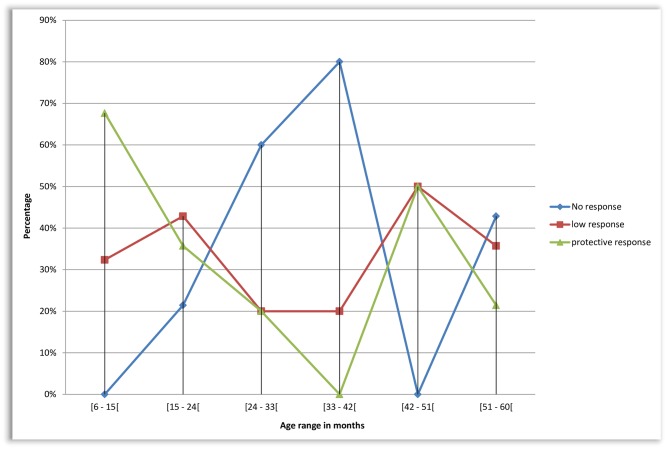
Trends of anti-HBV vaccine response by age. “Protective response” (Anti HB_S_ titer >10 UI/ml); “Low-response” (Anti HB_S_ titer 1–9 UI/ml); “No response” (Anti HB_S_ titer <1 UI/ml). Vaccine response is provided in 5 age groups, ranging from 6–59 months.

Evaluation of vaccine efficacy according to the age ranges of children revealed that protective-response decreased from 67.65% (23/34) for age 6–14 months, 35.71% (5/14) for age 15–23 months, 20% (1/5) for age 24–32 months, 0% (0/5) for age 33–41 months (Fig 2). Most importantly, protective-response was found in 58.33% (28/48) of children aged <24 months vs. 26.47% (9/34) ≥24 months, OR: 3.889 [CI_95%_ 1.362–11.356], p = 0.004; with specifically 67.65% (23/34) ≤6 months vs. 0%, (0/5) 33–41 months, p = 0.008.

Furthermore, among all HIV-exposed children (n = 54), age ≤12 vs. >12 months, respectively, confirmed the decreasing protection with higher age and HIV-infection: uninfected (71.4% [10/14] vs. 20% [3/15], p = 0.009) and infected (33.3% [2/6] vs. 15.8% [3/19], p = 0.562).

## Discussion

In highly endemic HBV and HIV settings like Cameroon, considering paediatric HIV-exposure when monitoring paediatric anti-HBV vaccine response might be of great clinical and public health relevance, and supports optimal performance of the EPI [6,7,10–12]. The present study could therefore generate evidence toward a strengthen vaccination policy in general, and that of anti-HBV in particular [11,23]. Complete negativity to anti-HBc and HBsAg in this population of Cameroonian children supports low risk of HBV vertical transmission in typical tropical SSA settings [12–15].

Though the majority (80.49%) of children were showed a response to anti-HBs testing, a non-negligible rate (19.51%) failed completely, highlighting the necessity for routine evaluation of post-vaccination to either confirm efficacy or determine eligibility for re-vaccination [19–22]. This indication might become more relevant as only 45.12% of children developed a protective immune response, in line with previous findings among Cameroonian children (42.9–68.0%) [13,18] and those from other SSA countries [13,17,20,21]. However, one study conducted in Cameroon showed a very high prevalence of children (92%) with anti-HBs ≥10 IU/L against lower-protection (58%) in Senegal [17]. Since the EPI in Cameroon uses the same Zilbrix vaccine (DTwP-HBV-Hib combination), environmental/programmatic factors affecting site performance (supply chain, cold chain, health staff, etc), or vaccine-integrity overtime, may be accessed thoroughly [13,17,18]. Furthermore, Quinvaxem (another manufacturer’s DTwP-HBV-Hib vaccine combination) showed suboptimal efficacy (58%) in a similar tropical country, indicating possible impact of environmental conditions compared to higher response in the western world [16]. Altogether, current findings underscore the need of systematic anti-HBs biological monitoring for every vaccinated child living in a tropical setting, to ensure maximal protectiveness against HBV [13,17,18, 19–21].

Levels of anti-HBV protection dropped significantly for HIV-vertically exposed and/or infected children. This reflects the potential impact of maternal immunodeficiency on the child protectiveness [8,9], and could be applicable to other vaccine responses (diphtheria, tetanus, pertussis and *Haemophilus influenzae* type b, etc) as earlier addressed [9,19,20,24]. This is particularly true as HIV-infected children generally experienced immunodeficiency in such context (i.e. 375 median CD4 cells/mm3 from our findings). Thus, HIV-unexposed children would likely develop an effective vaccine response compared to their exposed/infected counterparts. As early postulated by Abramczuck et al. [24], maternal immunosuppression is therefore a key concept to consider in the frame of predictive loss in vertical transmission of immunity [8,22,25]. This implies cost-effective analysis on either implementing a systematic biological monitoring of anti-HIV vaccine response to all HIV-exposed children, or implementing a public health policy by a boosted anti-HBV vaccination for all HIV-vertically exposed children. Moreover, as our observations were recorded in a context where HIV- infected children are all receiving antiretroviral therapy and generally asymptomatic (WHO stages 1 and 2), anti-HBV vaccination becomes more concerning when dealing with cases of HIV infection (irrespective of clinical conditions) compared to their uninfected peers, as also supported by Irungu *et al*. in Kenya [21,22,26].

Though feeding options, especially exclusive breastfeeding, had no significant impact on anti-HBV vaccine response (p = 0.757) in our dataset, further investigations with higher sample size are needed on the impact of feeding option on anti-HBV vaccine response. This is because breast-feeding is generally known to positively modulate response to vaccines (measles, mumps, rubella) through significant developmental changes in Th-1 pipeline response (CD81 T cells, natural killer cells, and mitogen-induced IFNg), as compared to formula-fed infants [27]. This is also justifiable by Th2 pathway-prone of antigen used in anti-HBV vaccine [28].

The quality of response appears to be decreasing, as children grow older, thus supporting the fact that anti-HBV immunization stimulates higher production of Th2-type cytokines and higher levels of antigen-specific type 2 immunoglobulins in newborns than in adults [28]. Though median age varied according to the study groups, IQR and min-max generally overlap, suggesting a limited impact of age variability on outcomes between the three study groups. In this light, our observation thereby indicates the importance of early-age vaccination on the early development of infant immune system while stressing on the necessity to streamline the accurate paediatric age (≥2 years from our findings) for controlling response and/or re-boosting vaccination (by booster-dose) [19,22,28], especially among HIV-exposed, HIV-infected. This strategy could also be expanded to all children (irrespective of HIV exposure) above 2 years old [27,28]. The required cost for anti-HBs testing, as well as the eventual vaccine booster dose, should be furthered analyzed for possible public health approach and implementation for resource-limited settings [19–22]. Comsidering and the national EPI our study target (children aged 0–5 years) falls in the range of the national framework, which makes the current findings convenient for policy-implementation [12,18].

### Study limitations

The high rate of non-consenting mothers is the major limitations to our study, as this is directly related to the statistical power in the three arms. Non-consenting were due to (a) believe that vaccination automatically provide the required protection, (b) fear that the child sample could be used for other purpose beside informed research goals, (c) fear of phlebotomy, (d) undisclosed or unknown motivations. These indicate needs for counselling parents on the importance of controlling vaccine efficacy, as well as sensitizing health staff on implementing such measures in routine care, for programmatic effectiveness [21].

Higher sampling would have allowed closer age-match, gender-match, and wider immune status analysis (if CD4 were available in all the arms) with respect to anti-HBV vaccine response [22]. Lack of data on nutritional status also restricts the breath of our findings, suggesting further investigations in tropical settings (whereby climate, cold chain and supply systems might be concerning). Furthermore, assessing AgHBs variants and occult HBV infection would have provided in-depth evidence on the observed vaccine response [29,30].

## Conclusions

Despite high response to anti-HBV vaccination among Cameroonian children, protectiveness is poor in general (<50%), worse with HIV-exposure, and even worst with HIV-infection; and a decreasing response as children grow older. Strategies supporting early-age vaccination as well as implementing policies to monitoring post-vaccination response and cost-effective analysis of specific immunization algorithms for HIV vertically exposed- or infected-children would be of high public health benefit in resource-limited settings. These observations therefore merit wider sampling for confirmation towards a stronger policy-implementation.

## Supporting Information

S1 File HBV-POST-VACCINE RESPONSE-CHILDREN-CAMEROON. (ZIP)
